# A novel gross deletion in the progranulin gene in four subjects with frontotemporal dementia

**DOI:** 10.1007/s10072-026-09030-3

**Published:** 2026-04-04

**Authors:** M. Ricci, G. Di Fede, P. Caroppo, V. Aprea, C. Villa, A. Romeo, P. Tiraboschi, A. Marti, M. Grisoli, M. M. Piccione, S. Cimini, Giacomina Rossi

**Affiliations:** 1 Unit of Neurology 8 - Dementia and Degenerative Pathologies of Central Nervous System, Fondazione IRCCS Istituto Neurologico Carlo Besta, Milan, Italy; 2 Neurology Unit, Neuromotor & Rehabilitation Department, Azienda USL-IRCCS di Reggio Emilia, Reggio Emilia, Italy; 3 Neuroradiology Unit, Fondazione IRCCS Istituto Neurologico Carlo Besta, Milan, Italy

**Keywords:** Progranulin, Mutation, Deletion, Frontotemporal dementia, Haploinsufficiency, MLPA

## Abstract

**Background:**

Mutations in progranulin gene (*GRN*) are a major cause of frontotemporal dementia (FTD). Most reported pathogenic mutations are nonsense, frameshift, or splicing mutations, resulting in a premature stop codon, degradation of mutated mRNA and consequent protein haploinsufficiency.

**Objectives:**

In this study, we analysed four subjects with FTD who had low plasma progranulin levels but no mutation detectable by sequencing of *GRN*, to disclose the underlying genetic cause of disease.

**Methods:**

Multiplex ligation-dependent probe amplification (MLPA) method was applied to search for rearrangements in *GRN*. Region-specific polymerase chain reaction (PCR) and Sanger sequencing were performed to define the breakpoint. Quantitative real-time PCR (qRT-PCR) on *GRN* mRNA and haplotype sharing analysis were also performed.

**Results:**

MLPA revealed in all the subjects the same heterozygous deletion, and a possible common ancestor was suggested by haplotype sharing. PCR and sequencing allowed us to define the size of the deletion (3028 bp), that removes part of *GRN* promoter, exon 1 including the transcription start site and most of the intron 1, and the breakpoints. qRT-PCR showed reduced level of mRNA, confirming the pathological nature of the deletion.

**Conclusions:**

In this study, we described a *GRN* heterozygous gross deletion which removes the consensus sequences for transcription factors and the transcription start site, leading to a reduced levels of plasma progranulin. Our study indicates that *GRN* rearrangements, although not common, should be investigated in patients with FTD who show low plasma progranulin levels but no *GRN* mutations detectable by DNA sequencing.

**Supplementary Information:**

The online version contains supplementary material available at https://doi.org/10.1007/s10072-026-09030-3.

## Introduction

Frontotemporal dementia (FTD) is a neurodegenerative condition characterized by heterogeneous clinical presentations, such as behavioural variant (bvFTD), semantic dementia and non-fluent variant of primary progressive aphasia (nfvFTD), as well as heterogeneous neuropathological substrates due to depositions of different misfolded proteins. Familial history is present in 30%−50% of FTD cases, and autosomal dominant transmission pattern can reach 27% frequency [1], underlining the relevance of genetic basis of these disorders. The major causative genes are microtubule-associated protein tau (*MAPT*), progranulin (*GRN*) and *C9ORF72* genes, while other genes account for a smaller number of cases [2].

Progranulin is a growth factor involved in inflammation, wound healing and cancer [3], which has been recognized as having a neurotrophic and neuroprotective function in central nervous system: in fact, heterozygous loss of function mutations in *GRN* give rise to FTD due to protein haploinsufficiency [4]. Low plasma progranulin dosage almost invariably suggests the presence of a loss of function *GRN* mutation [5].

Most pathogenic *GRN* mutations are small deletions or insertions, splicing or nonsense mutations, which lead to the introduction of a STOP codon which in turn activates the nonsense–mediated decay system degrading the mutated mRNA and causing progranulin deficiency. *GRN* gross deletions have been reported only in a few cases: deletions encompassing a full exon or part of it [6]; nearly all exons [7, 8] and entire gene [9]. Deletions involving 5’-untranslated regions (5’-UTR) and intron 1 have also been reported [10].

Here we report four FTD cases with plasma progranulin levels below the reference cut-off (61,55 ng/ml) [5], strongly suggesting the presence of a *GRN* mutation. Sequencing of exons, exon/intron junctions and the whole promoter region did not identify any mutation. Multiplex ligation-dependent probe amplification (MLPA) analysis disclosed the same gross deletion in all patients. Our study indicates that *GRN* rearrangements, although rare, should be investigated in patients with FTD who show low plasma progranulin levels but no *GRN* mutations detectable by standard DNA sequencing.

## Patients and methods

### Clinical data

#### *Patient 1*

**Fig. 1:**
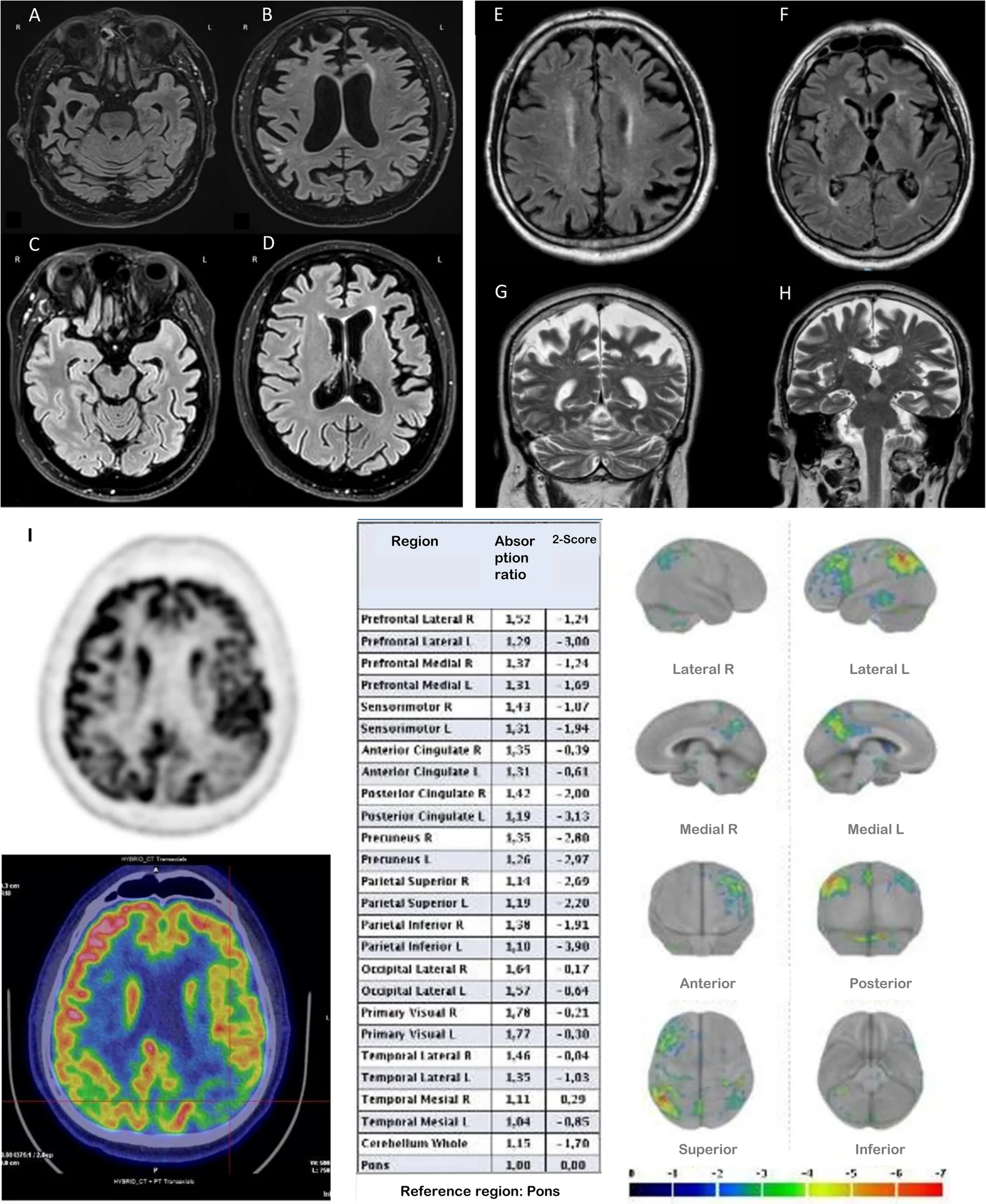
Imaging results. **A**, **B** Patient 1, proband: MRI FLAIR sequences show prominent right frontotemporal atrophy. **C**, **D** Patient 2, sister: MRI FLAIR sequences show slight left temporal atrophy and enlargement of left ventricle. **E**-**H** Patient 3: MRI axial FLAIR (**E**, **F**) and coronal T2-weighted sequences (**G**, **H**) show progression of cortical atrophy involving insula, frontal and bilateral parietal regions, more evident on the left. (**I**) Patient 3: Brain FDG-PET with qualitative evaluation and confirmation through statistical parametric evaluation, performed using CORTEX ID, showing hypometabolism in the left inferior parietal region and in the left lateral prefrontal cortex, posterior cingulate, and precuneus

The proband of family B developed, at the age of 68, memory impairment and verbal aggression, and later severe apathy. He also developed attention impairment, voracity and sweet food seeking. A neuropsychological evaluation revealed attentional-executive deficits and verbal comprehension and fluency impairments (Supplementary Table 1). Magnetic Resonance Imaging (MRI) revealed prominent right frontotemporal atrophy (Fig. 1A, B) and a Fluorodeoxyglucose-Positron Emission Tomography (FDG-PET) showed right orbitofrontal and dorsolateral hypometabolism. He was diagnosed with bvFTD, with prominent apathy, according to current diagnostic criteria proposed by proposed by Rascovsky et al. [11].

#### *Patient 2*

The proband’s sister developed, at the age of 57, memory impairment, temporo-spatial disorientation and mild expressive language disorder. MRI showed left temporal atrophy and enlargement of left ventricle (Fig. 1C, D) and the FDG-PET revealed decreased uptake in the left frontal, anterior cingulate regions, precuneus and inferior parietal lobule. The neuropsychological and behavioral evaluations showed attentional-executive deficits, verbal disinhibition, wandering and eating disorders (Suppl Table 1). She was thus diagnosed with bvFTD with prominent disinhibited/restless behavior [11].

Plasma level of progranulin was measured using an Enzyme-Linked Immunosorbent Assay kit (Adipogen), according to the manufacturer’s instructions. Plasma level of progranulin was 27,8 ng/ml in the proband and 28,4 ng/ml in his sister.

Family history was positive for neurodegenerative disorders, as the mother had been diagnosed with Parkinson’s disease at the age of 68.

#### *Patient 3*

The patient presented at age 58 with memory disturbances and a mild intentional and postural tremor of the left hand and head. Brain MRI showed unspecific vascular gliotic changes. The neuropsychological assessment revealed cognitive deficits involving attentional-executive functions, verbal long-term memory, and visuospatial learning (Supplementary Table 2). Depressive symptoms associated with anxiety and irritability were also present. Cerebrospinal fluid analysis of Alzheimer’s disease (AD) biomarkers was normal. He was diagnosed with amnestic multidomain mild cognitive impairment not due to AD.

At three-year follow-up, brain MRI revealed the presence of cortical atrophy predominantly involving left insula, frontal and, more markedly, bilateral parietal regions (Fig. 1E-H). Brain FDG-PET showed moderate-to-severe glucose hypometabolism in the left inferior parietal region and moderate involvement of the left lateral prefrontal cortex, posterior cingulate, and precuneus (Fig. 1I). Plasma progranulin level was reduced (37.6 ng/ml). The patient developed a severe non-fluent variant of primary progressive aphasia and was diagnosed as nfvFTD, according to the criteria proposed by Gorno-Tempini et al. [12]. Family history was notable for essential tremor on the paternal side.

Presently, after a seven-year follow-up, the patient is institutionalized, has lost functional independence and shows markedly impaired comprehension, mutism, postural and action tremor of the upper limbs, dysphagia for liquids, and hyperphagia.

#### *Patient 4*

The patient received an outpatient diagnosis of FTD; unfortunately, no additional clinical data are available. Progranulin dosage was 31,7 ng/ml.

### Genetic analysis

#### DNA sequencing

DNA was extracted from peripheral blood mononuclear cells (PBMC) using standard procedures. In addition to *GRN* coding exons and flanking intronic sequences (RefSeq NM_002087.4), full 5’- and 3’- untranslated regions and promoter region [13] were sequenced by Sanger technique.

As they were very recently examined, the two siblings of family B were also subjected to a new next generation sequencing custom panel of 77 causal and rare/risk genes associated with dementia (Supplementary Table 3); this panel was not investigated in the two unrelated subjects, as they had been visited a long time ago.

#### Multiplex ligation-dependent probe amplification (MLPA) analysis

To evaluate deletions or duplications of one or more exons of *GRN*, we performed MLPA using the SALSA MLPA Probemix P275-C4 MAPT-PGRN kit (MRC-Holland), following the manufacturer’s instructions. The amplification products were quantified using the Coffalyser.Net software. Details about this technique are reported in the Supplementary Data 1.

#### RNA analysis

We performed quantitative real-time PCR (qRT-PCR) using premade TaqMan gene expression assays to quantify *GRN* mRNA. Total RNA was isolated from PBMC of the proband, of a subject carrying the Asn118PhefsX4 *GRN* loss of function mutation (positive control) and of two wild-type *GRN* controls with the miRNeasy Mini Kit (Qiagen). cDNA was synthesized using High Capacity cDNA Reverse Transcription Kit and subjected to qRT-PCR using premade TaqMan gene expression assays: Hs00173570_ m1 for *GRN* and Hs99999903_ m1 for β-actin as an endogenous control for sample normalization (housekeeping gene) (Applied Biosystems). The cDNA was amplified in triplicate and run on an ViiA7 Real-time PCR system (Applied Biosystems). The relative transcription levels of *GRN* mRNA were calculated by using the 2^−ΔΔCt^ method, where ΔΔCt = ΔC_patient_−meanΔCt_controls_, and ΔCt=Ct_*GRN*_−Ct_β−actin_.

#### Haplotype sharing analysis

We performed a haplotype analysis using 8 short tandem repeat (STR) markers spanning a region of 7.29 Mb around *GRN* on chromosome 17q21. D17S1787, D17S1801, D17S951, D17S1861, D17S934, D17S950, D17S931 and D17S1827 were amplified with one fluorescently labeled primer and analyzed on a 3130xl Genetic Analyzer System (Applied Biosystems). Alleles were scored using the Peak Scanner Software (Thermo Fisher Scientific).

## Results

### Genetic analysis

#### DNA sequencing

*GRN* direct sequencing of the four subjects disclosed no mutations in coding exons, flanking intronic sequences, 5’ and 3’ untranslated regions nor in promoter region. Dementia gene panel in the two siblings was negative.

#### Multiplex ligation-dependent probe amplification (MLPA) and sequencing analyses

**Fig. 2:**
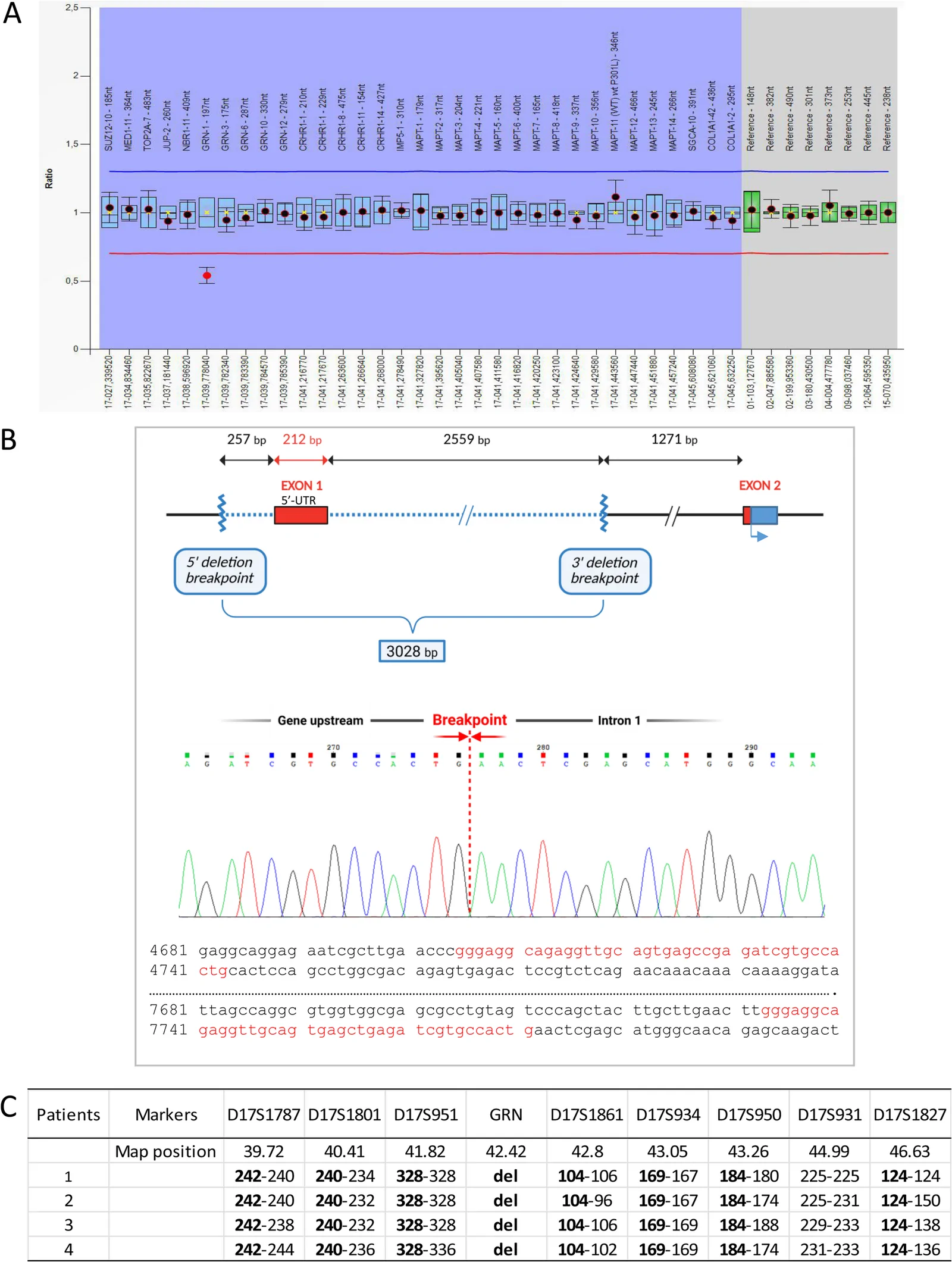
Genetic analyses. **A** Multiplex ligation-dependent probe amplification (MLPA) results showing the deletion of exon 1 in Proband. **B** Detailed representation of the region involved in the breakpoints, with the DNA sequence of two repeated regions (in red), presumably the recombination sites. The deleted region is: NG_007886.1:g.4744_7771del (chr17:42,422,234_chr17:42,425,261del). **C** Haplotype sharing analysis

MLPA analysis disclosed a deletion of exon 1 in all subjects (Fig. 2A and Supplementary Fig. 1). A series of PCR spanning the promoter region to the exon 2 (Supplementary Data 2) allowed us to exactly identify the DNA breakpoints (Fig. 2B): NCBI Reference Sequence NG_007886.1:g.4744_7771del (Human GRCh37/hg19, chr17:42,422,234_chr17:42,425,261del). The two short repeated sequences that may be the non-allelic homologous recombination sites are also indicated (Fig. 2B).

#### RNA analysis

Quantitative real-time PCR on *GRN* cDNA confirmed the reduction of mRNA to 53%, clearly suggesting that the transcription is prevented on the deleted allele: ΔCt±SD_wt−controls_=2.45 ± 0.28, ΔCt±SD_proband_=3.30 ± 0.41, ΔCt±SD_positive−control_=3.13 ± 0.27; 2^−ΔΔCt^ of proband = 0.53, 2^−ΔΔCt^ of positive control = 0.6; reduction for proband = 47%, reduction for positive control = 40%.

#### Haplotype sharing analysis

The haplotype analysis revealed that the four patients shared the alleles of 6 out of the 8 consecutive STR markers, spanning a 3.54 Mb region ranging from D17S1787 to D17S950 (Fig. 2C).

## Discussion

Since 2006 a large number of *GRN* mutations have been reported, accounting for approximately 5–10% of total FTD cases [14]. Most of the reported pathogenic *GRN* mutations are non-sense, splice-site mutations, as well as small insertions and deletions, while gross deletions were more rarely reported [7, 9, 10].

We disclosed a novel gross deletion in the progranulin gene in a subject affected by FTD, in the proband’s sister and in two apparently unrelated subjects. This deletion removes the final region of the *GRN* promoter, the non-coding exon 1 and most of intron 1.

Due to the resulting absence of consensus sequences for transcription factors and the transcription start site, transcription is reduced on the deleted allele, as evidenced by the reduction of *GRN* transcript and the low plasma progranulin level, thus confirming haploinsufficiency as the primary pathogenic mechanism underlying *GRN*-associated FTD [4].

Our patients were all native of Northern Italy and, due to the uniqueness of the mutation, we performed a haplotype sharing analysis, that suggested a possible inheritance of the same chromosomal trait of about 3,54 MB from a common ancestor.

Clinically, a high inter- and intrafamilial phenotypic variability is present in FTD cases associated with *GRN* mutations [15], possibly reflecting environmental or genetic [2] modifying factors. Indeed, the siblings of our study, although carrying the same deletion, showed different bvFTD phenotypes: the proband developed a more apathetic form of bvFTD, with prominent right-sided brain atrophy, whereas his sister presented with an earlier onset, more disinhibited and restless behaviour and predominant left-sided atrophy. Furthermore, patient 3 showed a different phenotype, consistent with nfvFTD.

## Conclusion

Our study highlights the need of investigating *GRN* rearrangements for a comprehensive genetic diagnosis in patients with low plasma progranulin levels but no conventional mutations. Hence the importance of a quantitative analysis such as MLPA to search for gross deletions/duplications that may explain the pathological loss of functional progranulin. An always updated knowledge of *GRN* variant landscape is essential, since therapeutic strategies targeting autosomal-dominant *GRN* mutations and aiming at raising progranulin level are emerging, such as gene replacement therapy using adeno-associated viruses, sortilin-targeted monoclonal antibodies, sortilin inhibitors, progranulin transfer [16].

## Supplementary Information

Below is the link to the electronic supplementary material.

Supplementary Material 1
