# Supplementary material for: A novel gross deletion in the progranulin gene in four subjects with frontotemporal dementia
Source: Neurol Sci. 2026 Apr 4;47(9):684. doi: 10.1007/s10072-026-09030-3 (PMC13447395; doi:10.1007/s10072-026-09030-3)
Supplement: Supplementary file 1 [file 10072_2026_9030_MOESM1_ESM.docx]

A novel gross deletion in the progranulin gene in four subjects with frontotemporal dementia

**Supplementary files**

**Supplementary Table 1.** Neuropsychological evaluation of Patient 1 and Patient 2

**Supplementary Table 2.** Neuropsychological evaluation of Patient 3

**Supplementary Table 3.** Gene panel sequenced by Next Generation Sequencing

**Supplementary Data 1**. MLPA Methodology

**Supplementary Figure 1.** Multiplex ligation-dependent probe amplification (MLPA) results for Patient 2, Patient 3 and Patient 4

**Supplementary Data 2.** Primers used to characterize the deletion region

**Supplementary Table 1**. Neuropsychological evaluation of Patient 1 and Patient 2

| **COGNITIVE DOMAINS** | **TEST** | **Proband** | **Sister** | **Cut-off** |
| --- | --- | --- | --- | --- |
| **Global cognition** | MMSE (Measso, 1993) | 14,85** | 19,31** | < 23,8 |
|  | CDT (Mondini, 2003) | 5** | 7 | 6 |
| **Language** |  |  |  |  |
| Phonemic and semantic based word retrieval | Letter fluency task (Novelli et al. 1986) | 8** | 2,2** | < 17 |
|  | Category fluency task (Novelli et al. 1986) | 12** | 13** | < 23,58 |
| Confrontational naming | CaGi (denomination) (Catricalà et al., 2013) | 46,05 | 42,16* | ≤ 41,48 |
| Comprehension | Token Test (Spinnler & Tognoni 1987) | 23,5** | 24** | < 26,5 |
| **Memory** |  |  |  |  |
| Working memory | Digit span forward (Monaco et al., 2013) | 3,99** | 3,77** | < 4,26 |
|  | Digit span backward (Monaco et al., 2013) | - | 1,66** | < 2,65 |
| Verbal episodic memory | RAVLT (Carlesimo et al., 1995) |  |  |  |
|  | -immediate recall | 27,3** | 12,8** | < 28,53 |
|  | -delayed recall | 5,8 | 0** | < 4,69 |
|  | -recognition | 11/15 | 7/15 |  |
|  | -false positive | 13 | - |  |
| Visuo-spatial memory | Corsi span (Monaco et al., 2013) | 2,94** | 3,73* | < 3,46 |
|  | Rey-Osterrieth Complex figure recall (Caffarra et al., 2002) | 12,5 | 3,5** | < 9,46 |
| **Attention and executive functions** | | | | |
| Executive abilities | FAB (Appollonio et al.2005) | 3.4** | 10** | ≤ 13,4 |
| Set-shifting/Attention | TMT B (Giovagnoli et al., 1996) | Int. | NE | < 282 |
|  | TMT B-A (Giovagnoli et al., 1996) | - | - | < 186 |
|  | TMT A (Giovagnoli et al., 1996) | 144** | 64 | < 93 |
|  | Attentional matrices (Spinnler & Tognoni, 1987*)* | 13,75** | 14,5** | ≤ 30 |
| **Visuo-spatial abilities** |  |  |  |  |
| Visuo-spatial constructional ability | Rey-Osterrieth Complex Figure copy (Caffarra et al., 2002) | 26,5* | 33,5 | < 28,87 |
| **Neuropsychiatrics symptoms** | | | | |
|  | NPI (Cummings et al., 1994) | 34 | 16 |  |
|  | FBI (Alberici et al., 2007) | 28/72 | 13/72 |  |
|  | FBI A | 21/36 | 10/36 |  |
|  | FBI B | 7/36 | 3/36 |  |

**Legend:** Neuropsychological evaluation (adjusted scores listed). * borderline, ** under cut-off, NE= not executable, Int.= Interrupted

**Supplementary Table 2**. Neuropsychological evaluation of Patient 3

|  | **range** | **N.V.** | **RS** | **CS** | **ES** |  |
| --- | --- | --- | --- | --- | --- | --- |
| **Neuropsychological Screening Test** |  |  |  |  |  |  |
| Mini-Mental State Exame (Measso, 1993) | 0-30 | ≥23,8 | **29** | **28,19** |  |  |
| MMSE Orientation | 0-10 | ≥6 | **9** |  |  |  |
| **Logical–Intellectual Functions** |  |  |  |  |  |  |
| Raven’s Coloured Progressive Matrices – CPM 47 (Carlesimo, 1995) | 0-36 | ≥18,96 | **32** | **30,70** | **4** |  |
| **Attention and Working Memory** |  |  |  |  |  |  |
| Visual Attention Test (Della Sala,1992) | 0-50 | ≥24 | **47** | **41,62** | **4** |  |
| Stroop Test – Short Version (Caffarra, 2002) |  |  |  |  |  |  |
| Word Reading (W) – seconds | … | ≤38 | **9** |  |  |  |
| errors | 0-30 | ≤2 | **0** |  |  |  |
| Color Naming (C) – seconds | … | ≤35 | **18** |  |  |  |
| errors | 0-30 | ≤1 | **0** |  |  |  |
| Interference (CW) – seconds | … | ≤80 | **56** |  |  |  |
| errors | 0-30 | ≤9 | **2** |  |  |  |
| Interference Effect – Time | … | ≤36,91 | **42,5** | **39,95** | **0** | ***** |
| Interference Effect – Errors | 0-30 | ≤4,23 | **2** | **1,80** | **2** |  |
| Trail Making Test (Giovagnoli, 1996) |  |  |  |  |  |  |
| TMT A seconds | … | ≤93 | **26** | **17,60** | **4** |  |
| TMT B seconds | … | ≤282 | **326** | **302,00** | **0** | ***** |
| B-A seconds | … | ≤186 | **300** | **284,40** | **0** | ***** |
| **Short-Term Memory** |  |  |  |  |  |  |
| Span (Monaco et al., 2012) |  |  |  |  |  |  |
| Digit Span Forward | 0-9 | ≥4,26 | **5** | **4,90** | **2** |  |
| Digit Span Backward | 0-8 | ≥2,65 | **3** | **2,85** | **1** |  |
| Digit Span Ratio | 0-1 | ≥0,48 | **0,60** | **0,60** |  |  |
| Corsi Span Forward | 0-8 | ≥3,46 | **4** | **3,88** | **1** |  |
| Corsi Span Backward | 0-9 | ≥3,17 | **4** | **3,99** | **2** |  |
|  |  |  |  |  |  |  |
| Corsi Span Ratio | 0-1 | ≥0,53 | **1,00** | **1,00** |  |  |
|  | **range** | **N.V.** | **RS** | **CS** | **ES** |  |
| **Long-Term Verbal Memory** |  |  |  |  |  |  |
| Babcock Story Recall (Novelli, 1986) |  |  |  |  |  |  |
| Immediate Recall | 0-28 |  | **9** |  |  |  |
| Delayed Recall (10') | 0-28 |  | **12** |  |  |  |
| Mean Number of Recalled Elements | 0-28 | ≥8 | **10,5** | **11,20** | **2** |  |
| 16 Unrelated Words Test (Mauri, 1997) |  |  |  |  |  |  |
| 1° Immediate Recall | 0-16 |  | **4** |  |  |  |
| 2° Immediate Recall | 0-16 |  | **5** |  |  |  |
| 3° Immediate Recall | 0-16 |  | **7** |  |  |  |
| 4° Immediate Recall | 0-16 |  | **5** |  |  |  |
| 5° Immediate Recall | 0-16 |  | **6** |  |  |  |
| Total Immediate Recall | 0-80 | ≥25,14 | **27** | **26,40** | **1** |  |
| Intrusions in Immediate Recall | … | ≤8 | **0** | **0,00** | **4** |  |
| Delayed Recall (15') | 0-16 | ≥3,44 | **3** | **2,94** | **0** | ***** |
| Intrusions in Delayed Recall | … | ≤3 | **0** | **0,00** | **4** |  |
| Forgetting Index | 0-100 | ≤ 60,56 | **50%** | **50%** | **1** |  |
| Recognition (30') | 0-32 | ≥22,02 | **29** | **28,74** | **3** |  |
| **Long-Term Visuo-Spatial Memory** |  |  |  |  |  |  |
| Supraspan Spatial Learning Test (Spinnler, 1987) |  |  |  |  |  |  |
| Immediate Recall | 0-29,16 | ≥5,75 | **4,34** | **0,29** | **0** | ***** |
| Delayed Recall (5') | 0-1,62 |  | **0,96** |  |  |  |
| Rey–Osterrieth Complex Figure (A) – Delayed Recall (10')(Caffarra, 2002) | 0-36 | ≥9,47 | **20** | **19,05** | **4** |  |
| **Constructional Praxis** |  |  |  |  |  |  |
| Rey–Osterrieth Complex Figure (A) – Copy (Caffarra, 2002) | 0-36 | ≥28,88 | **35** | **35,15** | **4** |  |
| **Visuo-Spatial Functions** |  |  |  |  |  |  |
| Benton Line Orientation Test – Form H | 0-30 | ≥18 | **23** | **24,00** | **3** |  |
| Clock Drawing Test (Mondini, 2003) |  |  |  |  |  |  |
| A. Numbers | 0-4 |  | **4** |  |  |  |
| B. Spatial Arrangement | 0-3 |  | **3** |  |  |  |
| C. Hands | 0-3 |  | **2,5** |  |  |  |
| Total Score | 0-10 | ≥7 | **9,5** |  |  |  |
| **Language** |  |  |  |  |  |  |
| Alternating Fluencies (Costa et al., 2014) |  |  |  |  |  |  |
| Phonemic Fluency (FAS) | … | ≥17,77 | **30** | **30,34** | **3** |  |
| Semantic Fluency (Colors / Animals / Fruits) | … | ≥28,34 | **46** | **48,13** | **4** |  |
| Alternating Fluency (A–Colors / F–Animals / S–Fruits) | … | ≥12,7 | **23** | **24,38** | **3** |  |
| Composite Alternation Index (CAI) | … | ≥0,38 | **0,61** | **0,62** | **2** |  |
| Visual Naming Test (Sartori, 1988, 1992): |  |  |  |  |  |  |
| Inanimate Objects | 0-32 | ≥28,03 | **32** |  |  |  |
| Living Things | 0-32 | ≥27,16 | **31** |  |  |  |
| Total Score | 0-64 | ≥56,21 | **63** |  |  |  |
| **Executive Functions** |  |  |  |  |  |  |
| FAB - Frontal Assess. Battery (Appollonio, 2005) | 0-18 | ≥13,5 | **13** | **12,46** | **0** | ***** |
| CET - Cognitive Estimation Task (DellaSala, 2003) |  |  |  |  |  |  |
| Total Errors | 0-42 | ≤18 | **12** | **12,97** |  |  |
| Total Bizarre Responses | 0-21 | ≤5 | **2** | **2** |  |  |

**Legend:** Normative Values (NV); Raw Score (RS); Corrected Score (CS); Equivalent Score (ES), * under cut-off

**Supplementary Table 3.** Gene panel sequenced by Next Generation sequencing

| ABCA1 | CLCN6 | GRN | PFN1 | SORL1 | UNC5C |
| --- | --- | --- | --- | --- | --- |
| ABCA7 | CLU | GSN | PICALM | SORT1 | VCP |
| ADAM10 | CSF1R | hnRNPA1 | PINK1 | SQSTM1 |  |
| AKAP9 | CST3 | hnRNPA2B1 | PLD3 | STH |  |
| APOE | CTSF | ITM2B | PRKAR1B | STX1B |  |
| APP | CXCR4 | KIF5A | PRKN | TARDBP |  |
| BCL7C | CYLD | LRP10 | PRNP | TBK1 |  |
| BIN1 | DCTN1 | LRRK2 | PSEN1 | TIA1 |  |
| CALHM1 | EIF4G1 | MAPT | PSEN2 | TMEM106B |  |
| CCL2 | EPHA1 | NCSTN | SCARB2 | TREM2 |  |
| CCNF | FLNC | NOS3 | SERPINI1 | TSC1 |  |
| CD2AP | FUS | NOTCH3 | SIGMAR1 | TTR |  |
| CD33 | GABRB3 | NPC1 | SNCA | TUBA4A |  |
| CHCHD10 | GBA | NPC2 | SNCB | TYROBP |  |
| CHMP2B | GIGYF2 | OPTN | SOD1 | UBQLN2 |  |

The analysis was performed with the Nextera Flex for enrichment kit (Illumina) coupled with gene-specific probes (Integrated DNA Technologies) on the MiSeq instrument (Illumina). MiSeq Reporter software (Illumina) was used for alignment (reference human genome UCSC hg19) and DNA variants were analyzed with Variant Studio software (Illumina), in addition to an in-home made bioinformatics pipeline.

**Supplementary Data 1**

**MLPA Methodology**

We performed MLPA using the SALSA MLPA Probemix P275-C4 MAPT-GRN (MRC-Holland), in combination with SALSA MLPA reagent kit and Coffalyser.Net data analysis software for the detection of deletions or duplications in the *GRN* gene.

In the first step, purified sample DNA is denatured. This is followed by overnight incubation with MLPA probes (including 5 probes for exons 1, 3, 6, 10 and 12 of the *GRN* gene), each consisting of a left and a right probe oligonucleotide (LPO and RPO) which hybridise to immediately adjacent target DNA sequences. After a ligation reaction, only possible for adjacent LPO and RPO probes, these probes are amplified in a multiplex PCR using a single universal primer pair. Only ligated probes are exponentially amplified. PCR products are then separated by capillary elettrophoresis; each fragment corresponds to a specific MLPA probe. Ligation and amplification of adjacent probes are prevented by deletion or insertion. Relative copy numbers of genomic targets are determined by comparing the resulting peak pattern of the test sample to that of a set of reference samples with a known normal copy number.

**A**

**B**

**B**

**C**

**C**

**Supplementary Figure 1. Multiplex ligation-dependent probe amplification (MLPA)** results show the deletion of exon 1 in Patient 2 (A), Patient 3 (B) and Patient 4 (C).

**Supplementary Data 2**

**Primers used to characterize the deletion region**

Primers for PCR amplification:

GRN-F: 5’-CTAGCTCCAGGCTTAGAACC-3’

GRN-R: 5’-CAGCTGCACCTGATCTTTGG-3’

Primer for sequencing:

GRN-Fseq: 5’- CTATGATCAGCTTGGGCGAC-3’
